# Evidence for Human Fronto-Central Gamma Activity during Long-Term Memory Encoding of Word Sequences

**DOI:** 10.1371/journal.pone.0021356

**Published:** 2011-06-29

**Authors:** Esther Berendina Meeuwissen, Atsuko Takashima, Guillén Fernández, Ole Jensen

**Affiliations:** 1 Donders Institute for Brain, Cognition and Behaviour, Radboud University Nijmegen, Nijmegen, The Netherlands; 2 Koninklijke Kentalis, Sint-Michielsgestel, The Netherlands; University of Bern, Switzerland

## Abstract

Although human gamma activity (30–80 Hz) associated with visual processing is often reported, it is not clear to what extend gamma activity can be reliably detected non-invasively from frontal areas during complex cognitive tasks such as long term memory (LTM) formation. We conducted a memory experiment composed of 35 blocks each having three parts: LTM encoding, working memory (WM) maintenance and LTM retrieval. In the LTM encoding and WM maintenance parts, participants had to respectively encode or maintain the order of three sequentially presented words. During LTM retrieval subjects had to reproduce these sequences. Using magnetoencephalography (MEG) we identified significant differences in the gamma and beta activity. Robust gamma activity (55–65 Hz) in left BA6 (supplementary motor area (SMA)/pre-SMA) was stronger during LTM rehearsal than during WM maintenance. The gamma activity was sustained throughout the 3.4 s rehearsal period during which a fixation cross was presented. Importantly, the difference in gamma band activity correlated with memory performance over subjects. Further we observed a weak gamma power difference in left BA6 during the first half of the LTM rehearsal interval larger for successfully than unsuccessfully reproduced word triplets. In the beta band, we found a power decrease in left anterior regions during LTM rehearsal compared to WM maintenance. Also this suppression of beta power correlated with memory performance over subjects. Our findings show that an extended network of brain areas, characterized by oscillatory activity in different frequency bands, supports the encoding of word sequences in LTM. Gamma band activity in BA6 possibly reflects memory processes associated with language and timing, and suppression of beta activity at left frontal sensors is likely to reflect the release of inhibition directly associated with the engagement of language functions.

## Introduction

Numerous electrophysiological studies point to oscillatory gamma activity playing an important role for neuronal processing [1], [2], [3]. Task dependent gamma activity has been reported during various types of cognitive processes including attention, motor planning, visual processing, working memory (WM) and long-term memory (LTM) [2], [4], [5], [6], [7], [8]. Long-term memory formation is often studied using subsequent memory paradigms in which the brain activity is compared for later remembered and later forgotten items. Subsequent memory effects in the gamma band have been observed during both encoding and retrieval in humans [5], [9], [10], [11], [12]. Oscillatory gamma activity might be particularly conducive to long-term formation since synchronized neuronal spiking promotes synaptic plasticity [13]. Furthermore, neuronal spiking phase-locked to the gamma activity has been shown to enhance synaptic efficacy [14]. Most of the gamma sources being modulated by LTM processing have been identified in posterior regions and the hippocampus [10], [11], [15]. Nevertheless, numerous fMRI and PET studies suggest that regions beyond the hippocampus and posterior brain regions play an important role for LTM processing [16], [17], [18]. In this study we have investigated whether gamma activity related to LTM can be identified in frontal regions.

We have applied a LTM task in which the presentation of the memory items (encoding) and the memory rehearsal are separated in time. This allowed us to investigate rehearsal related activity not contaminated by visual input. The task was composed of separate LTM and WM trials. In the LTM trials, subjects were instructed to remember the order of three words for later retrieval. In the WM trials subjects had to maintain the word order in the triplets for a short period. Processing related to LTM formation was identified using the comparisons LTM *versus* WM trials and later remembered *versus* later forgotten LTM trials. We measured ongoing brain activity with a whole-head MEG (magnetoencephalography) system to understand how oscillatory electrophysiological activity is modulated during memory formation and to identify the respective neuronal sources [19].

## Methods

### Participants

Twenty-five participants (14 females, 11 males; 18–27 years old) participated in this study. All participants were right handed, native Dutch speakers and had no history of neurological or psychiatric disorders including dyslexia (based on self reports). Datasets from two subjects were excluded from the analysis because of excessive head movements.

### Ethics statement

This study is approved by the ethical committee (Comissie Mens Gebonden Onderzoek Regio Arnhem-Nijmegen). Subjects gave written informed consent to participate in this study.

### Experimental design

**Figure 1:**
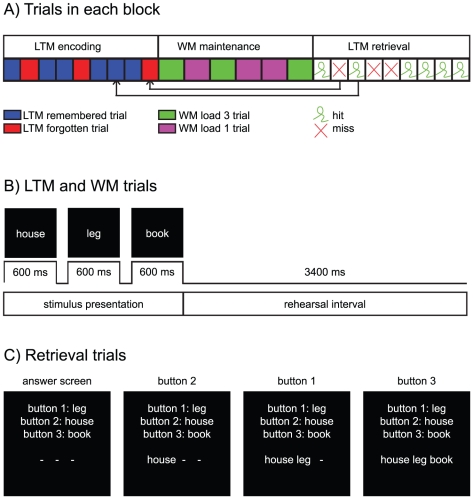
The paradigm. A) The task was composed of 35 blocks with each three parts: LTM encoding/rehearsal (9 or 11 trials), WM maintenance (6 or 7 trials) and LTM retrieval. B) In the LTM and WM trials, three words were presented sequentially (0.6 s/word) followed by a rehearsal interval of 3.4 s. C) In retrieval trials, participants reconstructed the word sequences learned during LTM encoding. Three words were shown and each represented by a button. Participants were asked to reproduce the initial order of the words by pressing the buttons in the correct order.

The task contained 35 (or 29) blocks and each block had three parts. Below, we will explain the criteria used to decide which version (35 or 29 blocks) was chosen. Each block started with 9 (or 11) LTM encoding trials, followed by 6 (or 7) WM maintenance trials and ended with 9 (or 11) LTM retrieval trials (Figure 1A). In LTM encoding and WM maintenance trials, three words were presented (600 ms/word) followed by a 3400 ms rehearsal interval (Figure 1B). Each word was used only once in the task (for a detailed description of the stimuli see [20]).

During the LTM encoding trials subjects were instructed to encode the order of the words in the triplet during the 3.4 s rehearsal interval. Subjects were aware that they would be asked to reproduce the word order later. The subjects' ability to immediately reproduce the word order in the triplets was tested in 20% of the trials (this was only done to make the LTM encoding part equal to the WM part). Trials followed by a test were excluded from the analysis.

During the WM maintenance trials, the presented word-triplet was to be maintained for 3.4 s. The triplets were composed of three different words (load three) or one word repeated three times (load one). Subjects' ability to reproduce the word order in the triplets was tested in 20% of the trials. For load-one trials, the same or a lure word was shown three times and subjects were asked to give a “match/no match” response. Trials followed by a test were excluded from the analysis.

During LTM retrieval trials, participants were asked to reconstruct the order of the words in the triplets they learned in the encoding part of the same block (Figure 1C). Every word was represented by a button. By pressing the buttons in the right order, subjects had to reproduce the learned sequences. Additionally, 20% catch trials were included in the retrieval part in which one of the words was replaced by a word not belonging to the triplet. When noticing these trials, participants had to press button 4.

To ensure an appropriate balance between the number of later remembered and forgotten trials for every subject, two versions of the experiment were made. One version contained 9 LTM sequences per block and 35 blocks in total, the other 11 LTM sequences per block and 29 blocks in total. As determined by their performance during the training session, six participants performed the 11-sequence task and the remaining 19 subjects the 9-sequence task. We aimed to get approximately 70% correctly retrieved sequences for each participant. After analyzing the data we checked whether the two groups of subjects showed similar effects in the gamma and beta band and that was indeed the case.

### Procedure

Participants visited the laboratory two times at successive days. The task was explained and practiced on the first day. To keep the encoding strategies similar for every subject, we encouraged subjects to construct sentences using the three words in each triplet.

On the second day the subjects performed the task in the MEG. Brain activity was recorded with a 275 axial gradiometer MEG system (VSM/CTF systems, Port Coquitlam, Canada) in supine position. The data were sampled at 1200 Hz and low pass filtered at ∼250 Hz. In addition, the horizontal and vertical electrooculograms (EOGs) were recorded to remove the effects of eye movements and blinks later during the offline preprocessing. The electrocardiogram (ECG) was recorded to be able to remove cardiac artefacts from the data. Head position was monitored using three coils placed at the nasion and in both ear canals. The recording session lasted approximately two hours including a 15 minute break. After the MEG recordings, a questionnaire was administered to evaluate whether participants applied the strategy provided in the instructions.

Finally, an anatomical MRI scan was acquired using a 1.5T (Siemens, Magnetom Avanto) or a 3T (Siemens, Magnetom Trio) MRI scanner. Ear plugs containing oil with vitamin E were placed in the ear canals during MRI acquisition enabling us to realign the MEG source reconstructions and the subject specific structural MRI data.

### Data analysis

The MEG data were analyzed using Fieldtrip; a Matlab toolbox developed at the Donders Institute for Brain, Cognition and Behaviour (website: http://www.ru.nl/neuroimaging/fieldtrip). Trials contaminated with muscle or SQUID artefacts were rejected. The data was down-sampled to 600 Hz after applying a 150 Hz low pass filter. Eye and heart beat artefacts were removed from the data using independent component analysis (ICA). On average, the LTM remembered, LTM forgotten, WM load 3 and WM load 1 conditions contained 186 (range: 123–242), 107 (range: 50–170), 95 (range: 81–103) and 94 (range: 73–105) trials, respectively.

### Spectral analysis

Time-frequency representations of power (TFRs; 4–32 Hz and 50–120 Hz) based on a sliding time window (steps of 50 ms) were computed from data segments recorded during presentation of the words (2.2 s) and the rehearsal interval (3.4 s). Power values for horizontal and vertical components of the planar gradients were calculated and summed for each sensor using signals from the neighbouring sensors, thereby approximating the signal measured by MEG systems with planar gradiometers [21]. For the lower frequencies (4–32 Hz), we used an adaptive time window containing 4 cycles (i.e. ΔT = 4/f) and applied a Hanning taper resulting in adaptive spectral smoothing of Δf∼1/ΔT. A fixed time window of 200 ms is used to analyze the high frequency oscillations (50–120 Hz) in the data. We used three orthogonal Slepian tapers which resulted in a spectral smoothing of ∼10 Hz. Absolute differences of average power estimates across tapers between conditions are reported. Note that the power estimates are not baseline corrected. This is not needed since power values are absolute measures. Also, the task had a blocked design and therefore different difficulty-expectancies for the LTM and WM conditions could have caused differences in the baselines.

### Source analysis

A beamforming approach using an adaptive filtering technique (Dynamic Imaging of Coherent Sources, DICS) was applied to the data to identify the sources of the oscillatory activity [22]. This spatial filter is constructed from the cross-spectral density matrix and the lead field matrix. It passes activity optimally from the location of interest while attenuating all other activity in the data. Cross-spectral density matrices were obtained from the Fourier transformed data for both LTM and WM trials during the rehearsal interval. The subjects' lead field matrices were calculated from a realistically shaped single-shell description of the brain based on the individual anatomical MRI [23]. A similar head model was constructed from a template MRI. The subject specific and template head models in MNI (Montreal Neurological Institute) coordinates were divided into regular 1 cm three-dimensional grids. Each individual's MRI was warped to the template MRI using SPM2 (http://www.fil.ion.ucl.ac.uk/spm) and the inverse of that warp was applied to the template grid. Because of this warping, a specific grid point is located at the same structural location in the template MRI and the subject specific anatomical MRIs. After applying the spatial filter to the data, the relative difference between the average power estimates of the two conditions was overlaid on the participants' MRI. Note that, for the source reconstruction, we used the data from the axial sensors and not the planar gradients.

### Statistical analysis

We applied statistical tests to the −1.7–0.0 s interval in which the words were presented and the 0.0–3.0 s interval in which subjects rehearsed the order of the words (t = 0 s indicates the start of rehearsal interval). A non-parametric cluster-based randomization test was applied to the sensor and source level data [24]. This test controls for type 1 errors in situations involving multiple comparisons by clustering neighbouring channels or grid points which show the same effect. If the t-value at a sensor/grid point exceeded a threshold (p<0.05) these sensors/grid points were included in a cluster. The cluster-level statistic of each cluster was defined as the sum of the t-values of all sensors/grid points in the cluster. The cluster with the maximum summed t-values was used as a test statistic and compared to the randomization null-distribution. To make the randomization null-distribution, per participant averages for both conditions were randomly divided in two groups and the maximum cluster-level statistics were calculated. This procedure was repeated for 500 times.

## Results

In this experiment, we investigated the oscillatory activity related to encoding and maintenance of word triplets in LTM and WM, respectively. The experiment was composed of LTM encoding trials, WM maintenance trials and LTM retrieval trials (Figure 1).

### Behavioral results

Triplets encoded in LTM were retrieved successfully in 64.6±10.2% of the trials (chance level = 16.6%). For WM load 3 and WM load 1 trials, subjects responded correctly in 91.6±7.2% and 97.3±4.0% of the trials, respectively. When evaluating which strategies subjects used, they reported to have used a rote rehearsal or no strategy for the WM trials (10 and 8 participants, respectively). During LTM trials, subjects often made sentences or had a combined visual and sentence making strategy (16 and 6 participants, respectively).

### Increased gamma power during encoding in LTM compared to maintenance in WM

**Figure 2:**
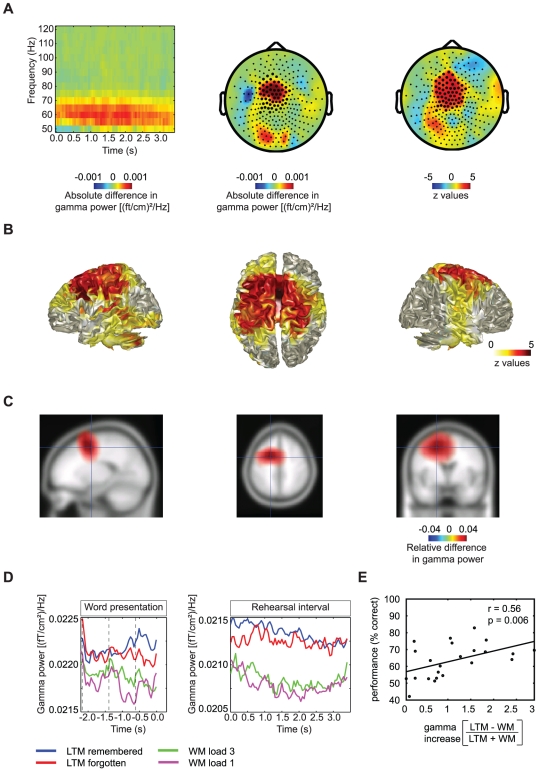
LTM−WM: effects in the gamma band. A) Time-frequency representation of the sensors in the significant cluster and topographic representations when comparing gamma band activity during the rehearsal interval of LTM encoding and WM maintenance trials. An increase in 55–65 Hz power can be observed at the marked sensors in the topographical plot. The z-values (right panel) show the differences normalized by variance. B,C) The beamformer analysis identifies the sources of the gamma band increase to the medial part of left BA6. Z-values of the statistical comparison (B) and power values between half of the maximum to the maximum value are shown (C). D) The average gamma power (55–65 Hz band) in the significant cluster of sensors is shown over time for all conditions separately during presentation of the words and in the rehearsal interval. E) There was a positive correlation over subjects between gamma power (LTM−WM/LTM+WM) and performance on the LTM task (r = 0.56, p = 0.006, N = 23).

Time-frequency representations (TFRs) of power were calculated for the rehearsal interval of LTM and WM trials. Comparing the oscillatory activity in these two trial types revealed a robust difference in gamma power (55–65 Hz) over left central sensors which was stronger in LTM encoding trials (p<0.0001, 0.0–3.0 s) (significant sensors are marked in Figure 2A). The change in gamma activity was sustained during the full rehearsal interval. A beamforming analysis was applied to identify the source of the difference in gamma power. The source appeared strongest over the medial part of left BA6 including large parts of the supplementary motor area (SMA) and pre-SMA. Figure 2B shows the positive z-values found when comparing relative difference in gamma power (55–65 Hz) between LTM and WM trials per grid point (p<0.0001). The average power obtained in the same analysis is shown in Figure 2C (threshold at half of the maximum). When performing time-frequency analysis on the data recorded during presentation of the words, we noticed that the difference in gamma power emerges even before the rehearsal interval begins (p<0.0001, −1.7–0.0 s). Figure 2D shows the temporal development of gamma power (55–65 Hz) during word presentation and the rehearsal interval for the sensors in the significant cluster (which are marked in Figure 2A).

### Subsequent memory effect: stronger gamma activity for later remembered compared to later forgotten trials

**Figure 3:**
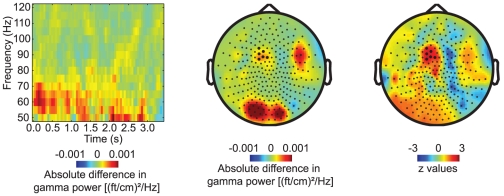
Subsequent memory effect in the gamma band. A subsequent memory effect was observed in the gamma band (55–65 Hz over left central sensors) when comparing later remembered to later forgotten LTM trials. The effect was significant when considering the first half of the retention interval and including only left fronto-central sensors. The right panel shows the difference in gamma activity when normalized with the variance (z-values) and confirms that the dominant effect is indeed at left central sensors.

Next, we investigated the subsequent memory effect during the rehearsal interval. The trials from the LTM encoding part were divided in two groups; triplets that were correctly and incorrectly reproduced during LTM retrieval trials (‘later remembered’ and ‘later forgotten’ trials, respectively). We observed an increase of fronto-central gamma band activity when comparing the activity for these two conditions (Figure 3). When applying a cluster based randomization test including all sensors, there was a weak trend (55–65 Hz, 0.0–3.0 s, p = 0.19). When we constrained the statistical comparison to the first half of the rehearsal interval and included only sensors in the significant cluster found when comparing LTM and WM trials, the subsequent memory effect was significant (p = 0.032). Like the difference in gamma activity between LTM and WM trials, this effect started to emerge at the end of the word presentation period (see Figure 2D). When considering the topography of the subsequent memory effect (Figure 3) differences over posterior regions and right frontal areas can be observed. Normalizing the difference with the variance (z-values) shows that these effects are driven by a few subjects and not strong enough to be significant on the whole group level.

### Decreased beta power during encoding in LTM compared to maintenance in WM

**Figure 4:**
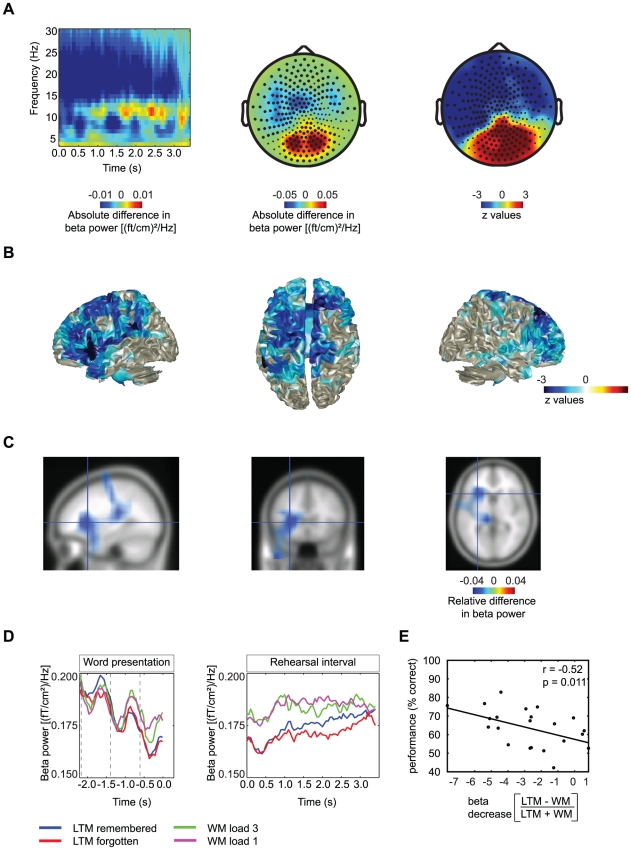
LTM−WM: effects in the beta band. A) Time-frequency representation of the sensors in the significant cluster and topographical representations of beta band activity when comparing the rehearsal interval of LTM encoding and WM maintenance trials. A decrease in beta power (15–27 Hz) is shown at sensors marked in the topographical plot. The topography of the z-values is shown in the most right panel. B,C) The sources of the decrease of beta power when comparing LTM encoding to WM maintenance. The sources of this effect are widespread but include LIFG and left insula. Z-values of the statistical comparison (B) and power values between half of the maximum to the maximum value are shown (C). D) The average beta power from sensors in the significant cluster shows the time-course of the effect for all conditions separately during word presentation and rehearsal. E) There was a negative correlation between beta power (LTM−WM/LTM+WM) and performance for each subject on the LTM retrieval test (r = −0.52, p = 0.011, N = 23).

Next, TFRs where calculated for lower frequencies (4–32 Hz). Results in the alpha band have already been reported in Meeuwissen et al. (2010) [20]. During the rehearsal interval beta power over anterior regions was significantly lower for LTM trials compared to WM trials (15–27 Hz, 0.0–3.0, p<0.0001) (Figure 4A). To find the dominant source of this effect, we applied a beamforming approach (Figure 4B,C). The relative difference in source level power was subjected to a cluster-randomization procedure (p<0.0001). Figure 4B shows the negative z-values projected to the brain surface. The effect is widespread but significant in left inferior frontal gyrus (LIFG) and the left insula (Figure 4C). When examining the time courses of beta power at significant sensors (which are marked in Figure 4A) for all conditions it becomes clear that beta power decreased with each word that was presented but was quite constant in the rehearsal period (Figure 4D). Besides a decrease in beta power at anterior sensors when comparing the rehearsal intervals in LTM and WM trials, we found a significant power increase in the beta band at posterior sensors (p = 0.004, Figure 4A).

### Gamma and beta power modulations correlate with LTM performance over subjects

**Figure 5:**
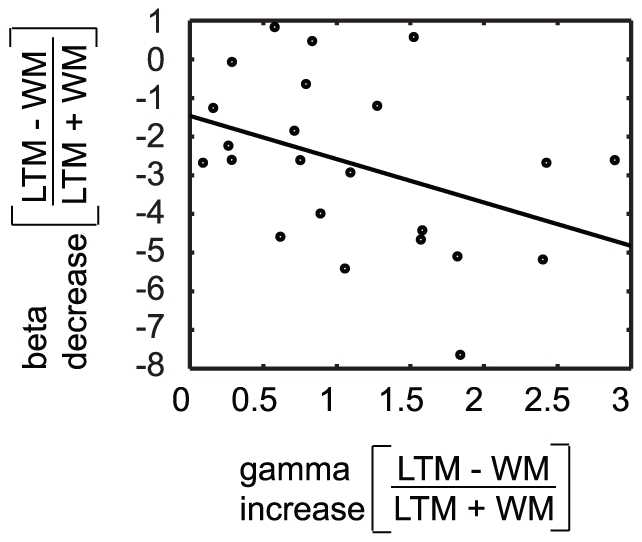
Correlation between gamma and beta power. Correlation between modulations of gamma and beta power at the sensors where the significant effects were found, over subjects (N = 23). A negative correlation was found between gamma and beta power modulations (r = −0.48, p = 0.022).

To substantiate that the gamma and beta modulations were related to processes important for memory encoding, we correlated the power changes with memory performance over subjects. The relative differences in gamma (55–65 Hz, 0.0–3.0 s) and beta power (15–27 Hz, 0.0–3.0 s) at sensors where the effect was significantly different between the LTM encoding and WM maintenance conditions (LTM−WM/LTM+WM) were calculated for all subjects and correlated to the hit rate on LTM trials. The gamma modulation correlated positively with the performance (r = 0.56, p = 0.006, Figure 2E) whereas the beta modulation correlated negatively with performance (r = −0.52, p = 0.011, Figure 4E). Next we correlated the beta and gamma power modulations directly with each other. We found a significant negative correlation (p = 0.022, r = −0.48, Figure 5). In sum, these findings show that individuals who are better at encoding the word sequences in LTM, are also individuals with larger power differences in the gamma and beta band when comparing LTM and WM trials.

## Discussion

In this study we set out to investigate oscillatory activity associated with LTM encoding and WM maintenance. We observed a sustained robust increase in gamma activity (55–65 Hz) over left fronto-central sensors during rehearsal to encode word triplets in LTM compared to rehearsal to maintain similar triplets in WM. The sources of the gamma band activity were located in the medial part of BA 6 (SMA and pre-SMA). This increase in gamma power correlated with LTM performance over subjects. Additionally we observed a weak subsequent memory effect: gamma power was stronger for later remembered compared to later forgotten trials. This effect was significant when constraining the statistical analysis to the first half of the retention interval and considering only the fronto-central sensors. To the best of our knowledge, this is the first study to report a robust gamma activity in fronto-central midline regions (pre-SMA and SMA) associated with LTM encoding. We did not find significant effects in the gamma band over posterior sensors. Furthermore, we found a power decrease in the beta band (15–27 Hz) over anterior sensors when comparing rehearsal intervals during LTM encoding and WM maintenance trials. The sources of this effect included LIFG and the left insula. The beta power decrease also correlated significantly with performance over subjects.

In animal studies modulations in neuronal synchronization in the gamma band have been demonstrated in various tasks [25], [26], [27], [28]. This has led to the proposal that gamma band activity plays an important role in neuronal computations. It has been less straight forward to identify gamma activity in humans. Gamma power modulations associated with cognitive processes in humans were first identified using EEG recordings [29], [30]. Interestingly, the first attempts to identify gamma activity with MEG failed [31] resulting in discussions about the reasons why MEG might be blind to gamma oscillations on a more physiological level [3]. Later, some of the EEG findings on gamma band activity were brought into question after it was discovered that micro-saccades can produce EEG artifacts in the gamma band [32]. However, a clear system gamma band activity has now been unequivocally demonstrated. For instance, Hoogenboom et al. [6] used MEG to demonstrate a clear sustained gamma activity produced by visual areas in response to moving gratings (other examples: [6], [7], [8], [33], [34], [35], [36]). Most of the studies reporting a robust, sustained, band-limited gamma band activity have identified sources in posterior regions. Despite the fact that the anterior part of the brain is heavily involved in cognitive processing, reports on robust and sustained frontal gamma activity identified with MEG and EEG are scarce [37], [38], [39]. We hypothesized that a demanding LTM memory task would engage frontal regions. In this study we provide evidence for robust, sustained and band-limited gamma band activity produced in the medial part of BA6. The presence of sustained gamma activity suggests that this region is more involved in rehearsal when encoding word sequences in LTM compared rehearsal to maintain these sequences in WM. The significant correlation between the gamma power increase and memory performance substantiates the interpretation that neuronal synchronization in the gamma band in BA6 is actively involved in LTM memory encoding.

The bilateral pre-motor cortex has been found to be associated with subsequent memory formation in numerous studies (for a review see Kim [18]). Which processes relevant for memory formation could have induced gamma activity in BA6? Various studies have demonstrated that BA6 is engaged in motor preparation [40] (such as the initiation and execution of speech), timing [41] and word production [42] processes. We can exclude preparation of button presses as the cause of the effects we found, because no button presses were required or made after the rehearsal interval. Several findings indicate that memory performance is improved when using an elaborative rehearsal strategy instead of a rote rehearsal or no strategy [43], [44], [45]. Also, a failure to process stimuli semantically lead to worse performance in an incidental LTM test [46]. In our study, subjects typically formed sentences in order to support LTM encoding, while most of them did not form sentences to maintain the word order in WM. Differences between these strategies used during LTM encoding and WM maintenance include (preparation of) sub-vocal speech and timing of the phonemes, words and punctuation in the sentence. Activity in BA6 is associated with these processes [42], [47]. Since previous studies have shown that elaborative encoding results in better performance, we suggest that the subjects with stronger frontal gamma power modulations and better performance are also the subjects that use a more elaborative encoding strategy.

Besides the modulation of gamma power, we also observed a modulation of beta power (15–27 Hz). Generally, beta power decreases when an area is engaged in the task [48], [49]. We found a suppression of beta power during the rehearsal interval of LTM compared to WM trials in anterior regions, and this effect was negatively correlated to performance. The sources reflecting the effect in the beta band were quite widespread but most prominent in the LIFG and the left insula. The result we report is similar to results described in Hanslymayr et al. [50]. They report a decrease in beta power over frontal sensors during deep semantic compared to shallow semantic encoding. When assuming that deeper semantic encoding took place during LTM than during WM trials this result fits well with our findings. Interestingly, beta power decreased systematically with the presentation of each word suggesting that the beta power decrease reflects processing of an increasing amount of semantic information. This result is in line with the hypothesis that activation of the left inferior prefrontal cortex reflects semantic working memory processes possibly relevant for memory formation [51]. More in general, a growing body of evidence implicates left prefrontal regions in language processes [42], [47], [52], [53]. Our results suggest that the left frontal activation is reflected by decreased power in the beta band.

In this MEG study, we have identified a part of a network involved in memory operations. A meta-analysis of studies investigating successful encoding in LTM showed that activity in the left inferior frontal cortex, fusiform gyrus, medial temporal lobe, premotor cortex and posterior parietal cortex contribute to successful storage of various types of information in LTM [18]. A similar network has been identified when comparing elaborative and rote rehearsal of verbal stimuli: BOLD activations related to elaborative rehearsal were observed in the LIFG, SMA/pre-SMA and the cerebellum [44], [45], [54], [55]. When considering brain activity during maintenance in WM of verbal compared to visual stimuli, these three areas appear more engaged as well [56]. Together these studies suggest that processes in the LIFG and premotor regions (SMA/pre-SMA) are important during several memory operations such as WM maintenance, rehearsal and LTM encoding. Our data add new insight by demonstrating that the engagement of the SMA/pre-SMA and LIFG/left insula are reflected by increased gamma band activity and decreased beta band activity, respectively. These effects correlated significantly with performance on the LTM retrieval trials which shows the relevance of these processes for successful encoding of word sequences,

Previously, we have reported a subsequent memory effect in the alpha band using the same data set [20]. We found that while the posterior alpha activity was suppressed during word presentation, it increased during the rehearsal interval. This increase was stronger during later remembered compared to later forgotten word triplets and stronger during LTM than WM trials. Figure 4A shows that the latter effect extends to the beta band. The posterior beta band effect is likely to be explained by harmonics in the alpha band. In line with other studies [57], [58], we argue that the posterior alpha band activity reflects a suppression of posterior regions in order to allocate neuronal resources to areas involved in the memory formation. The previous work and the results reported here together, suggest that LTM memory operations rely on an extended network in which some areas are engaged and others disengaged. Engagement and disengagement of the nodes in the network are reflected in different frequency bands. In future works, it would be important to uncover how these nodes communicate. One approach to do so is by applying measures of cross-frequency coupling.
